# Improving Intelligent Vehicle Control with a Prediction Model of Passenger Comfort Based on Postural Instability Parameters

**DOI:** 10.3390/s25175529

**Published:** 2025-09-05

**Authors:** Bin Xu, Hang Zhou, Yuanlong Zhou, Yunhao Wang, Zhen Li

**Affiliations:** School of Automobile, Chang’an University, Xi’an 710064, China; 2022022501@chd.edu.cn (B.X.); 2023222065@chd.edu.cn (Y.Z.); 2023222053@chd.edu.cn (Y.W.); lizhen@chd.edu.cn (Z.L.)

**Keywords:** passenger comfort, posture instability, intelligent vehicle, prediction model, attention mechanism

## Abstract

With the development of technology, comfort has gradually developed into the main criterion for evaluating intelligent vehicle performances. In this study, a field test was carried out under five common driving conditions, and 60 participants took part. Passenger posture data, vehicle motion data and passenger subjective comfort data were collected. A paired sample T-test and a ridge regression algorithm were used to explore the relationship between passenger posture swing parameters and subjective comfort. The results show that under the same driving conditions, the speed of posture swing was significantly higher for passengers who experienced discomfort. Furthermore, we found that the change in angular velocity was the main cause of passenger discomfort under different driving conditions. This suggested that the design of intelligent vehicle algorithms should focus on the angular velocity variation among passengers. Finally, based on traditional machine learning algorithms and deep learning algorithms, this paper establishes two models for predicting comfort through passenger posture instability. The accuracy of the machine learning model in predicting passenger comfort was 87.1%, while that for the deep learning model was 89%. The findings are useful in providing a theoretical basis for improving the comfort of intelligent vehicles.

## 1. Introduction

Intelligent driving vehicles can provide a convenient, efficient, and environmentally friendly way of travel. Fully automated vehicles can free people from heavy driving, allowing them to do whatever they want during the ride. At the same time, due to the changes in vehicle control (the driver becomes a passenger) and the impact that of people’s riding experience (people have less experience of riding in smart-driving vehicles) on motion sickness, emerging intelligent driving vehicles will create a riding experience that is more uncomfortable than that in a traditional vehicle [1,2,3,4]. However, the comfort of a ride is an important evaluation standard in travel services, and improving the ride comfort of intelligent driving vehicles can be beneficial to improving their popularity [5]. Therefore, current research has begun to focus on improving the comfort of intelligent driving vehicles.

Motion sickness is common in healthy people and is a general name for carsickness, seasickness, and other similar symptoms that can create severe discomfort. Early explanations of motion sickness utilized the theory of perception conflict [6,7]. The perception conflict theory states that the cause of motion sickness is the inconsistency a passenger experiences between his or her expected and actual perception. It was later found, however, that in the darkness, when the visual signal input is absent and thus there is no perceptual mismatch, people still experience dizziness. Therefore, Teixeira et al. [8] extended the postural-instability framework, defining posture control as the coordinated stability of all body segments and showing that reducing postural sway directly lowers cybersickness severity. Furthermore, Yang et al. [9] employed balance-training and moving-room–style paradigms to demonstrate that increases in postural instability reliably precede and predict the onset of motion-sickness symptoms.

Considering the uncomfortable experience of carsickness, researchers have performed a lot of work on how to alleviate carsickness. At present, the most widely used method is to use carsickness drugs. Although carsickness drugs can alleviate the symptoms of carsickness to a certain extent, they can also bring many side effects, including drowsiness and headache. Therefore, scholars began to try to reduce the possibility of car-sickness from the perspective of vehicles. Recent studies [10,11] indicate that vehicle control algorithms and motion planning can significantly reduce motion sickness by shaping acceleration profiles to avoid nauseogenic frequencies. Additionally, systematic reviews [12] have identified interior layout, vehicle dynamics, availability of anticipatory motion cues, visual access to road and non-driving related tasks as key factors influencing passenger comfort. Other human-centered design studies [13] have optimized seat-pan angle and posture ergonomics, demonstrating improved ride comfort through seat design modifications. For the traditional vehicle driven by human, the driver’s operation level is the influencing factor of vehicle comfort, while for the intelligent vehicle driven by algorithm, the automatic control algorithm is the key factor of passenger comfort.

The current intelligent control strategies for passenger comfort are mainly aimed at the control of excessive acceleration and deceleration caused by sudden braking and changes in direction, prompting researchers to establish a comfort threshold. Recent work [2] proposes lateral acceleration and jerk limits of approximately 0.84 m/s^2^ and 0.42 m/s^3^, respectively, as thresholds where discomfort begins in automated vehicles. Lai et al. [14] introduced a trajectory-planning based emergency braking control method that balances collision avoidance with passenger comfort, applying deceleration thresholds near 4–5 m/s^2^ to mitigate abrupt stops without compromising safety. Studies on car-following behavior in autonomous shuttles [15] show that higher jerk and deceleration values adversely impact ride comfort, suggesting more conservative deceleration and jerk limits in high-risk scenarios. However, in a fully intelligent vehicle, passengers may experience acceleration and directional changes in medium to high-risk scenarios. To ensure safety, vehicle motion parameters usually cannot meet the requirements of comfort thresholds. In addition, if the automatic vehicle control algorithm will be adjusted in this way, it will not be able to realize the value of improving the road capacity of autonomous vehicles [16]. Therefore, it is necessary to study the objective indicators of passenger comfort.

For many years, predicting passenger comfort has been challenging, and two main reasons contribute to this difficulty. First, there has been a lack of effective objective indices of comfort. Earlier research relied on physiological parameters such as electroencephalogram, heart rate, and blood pressure, which are difficult to collect and require further study to identify the dominant factors influencing motion sickness. Second, building a passenger comfort prediction model requires correlating subjective feelings with vehicle motion states or postural instability in real time. Traditional tools—from questionnaires to verbal-score scales like the Fast Motion Sickness Scale—did not allow for real-time data collection tied to specific vehicle motions, fundamentally limiting prediction capability. Recent studies are addressing both problems. Researchers now leverage physiological signals—such as ECG-derived heart rate variability (HRV), facial skin temperature, cerebral blood oxygenation, and posture data—to serve as objective indicators that reflect cognitive and comfort states during motion [17,18]. For example, Meng et al. [19] demonstrated that these physiological signals can reliably represent mental and emotional comfort levels of passengers in electric vehicles under real driving scenarios. Hwang et al. [20] measured physiological responses and subjective comfort in unstable autonomous driving conditions, enabling real-time correlation between motion state and subjective feedback. Building on these advancements, the present study aims to address a remaining critical challenge: the lack of tools for real-time acquisition of subjective comfort data. To this end, we designed a passenger comfort acquisition device was designed to realize the real-time acquisition of subjective comfort data, which lays the foundation for the prediction of the subjective comfort of passengers.

Although many efforts have been devoted to the improvement of the comfort of intelligent driving vehicles, there currently is no effective passenger comfort prediction model, thereby making it difficult to make a major breakthrough in this field. In this study, we clarified the mechanism of passenger discomfort by analyzing the relationship between the passenger’s postural instability parameters and the subjective comfort, and finally established a passenger comfort prediction model. This research can provide a reference for the development of intelligent driving vehicle motion control algorithms, and for strategies that consider comfort while aiming at improving the public acceptance of intelligent driving vehicles.

## 2. Method

Although autonomous vehicles are already being tested on public roads, it is still difficult to use them for comfort tests due to safety reasons and the complexity of the test algorithms. It is more effective to use experienced human drivers and traditional vehicles to simulate the typical driving conditions of the driverless vehicles [21,22].

### 2.1. Participants

Sixty participants took part in this study, with a balance of male and female participants. Participants’ ages ranged from 21 to 28 years (Mean = 24.3, Standard Deviation = 3.8). We selected the 21–28 yr age range to ensure at least three years of driving experience (reducing novice risk-taking variability), target the life-stage with peak motion-sickness susceptibility and inter-subject variability [6], and control age-related vestibular/sensorimotor changes that might confound posture-instability measures [23].

Through analysis of participants’ demographic characteristics and health screening, all participants were confirmed to meet the following criteria: a body mass index (BMI) within the normal range of 18.5–25 kg/m^2^; no history of musculoskeletal diseases (e.g., neck pain, back pain), vestibular system disorders, or other chronic conditions that might affect posture; and all reported as being in a healthy state via a pre-test health self-assessment questionnaire.

According to the motion sickness susceptibility questionnaire (MSSQ) [24] filled out by the participants, they were divided into two groups: sensitive to motion sickness and not sensitive to motion sickness. Participants were divided equally into four groups based on gender and susceptibility to motion sickness, and fifteen participants in each group.

A skilled driver with 13 years of driving experience and no traffic accidents in the past five years was recruited as the driver for this test. As a participant in this research, the driver fully understood the purpose and route of the experiment.

The driver was trained on the test track operation for two days before the test to ensure that the designated operations could be completed as required.

### 2.2. Experimental Equipment

**Figure 1:**
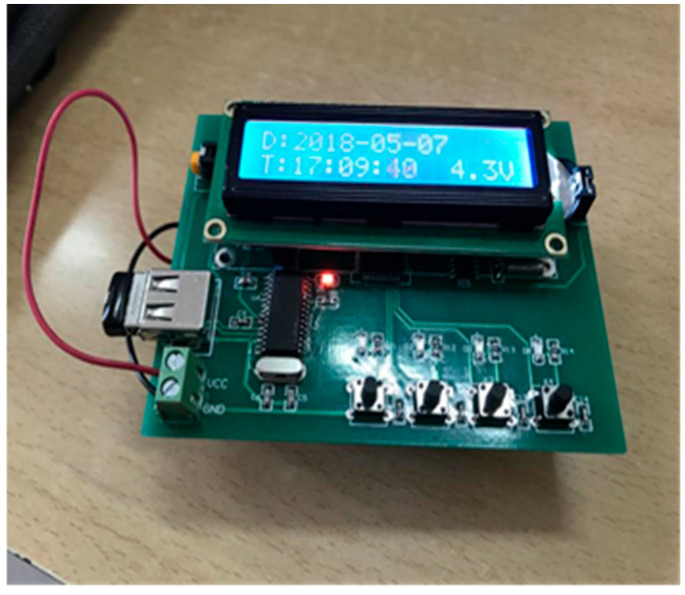
Handheld rating device.

This study was conducted using an automatic-transmission passenger car, given that a gear shift in a manual-transmission vehicle may affect passenger comfort [25]. Participants used a handheld rating device (Figure 1) composed of a screen, four buttons, a data storage unit, and a power supply device to evaluate their comfort. The rating device recorded the participants’ dichotomous responses of ‘no-discomfort’ or ‘discomfort’ without affecting their postures.

**Figure 2:**
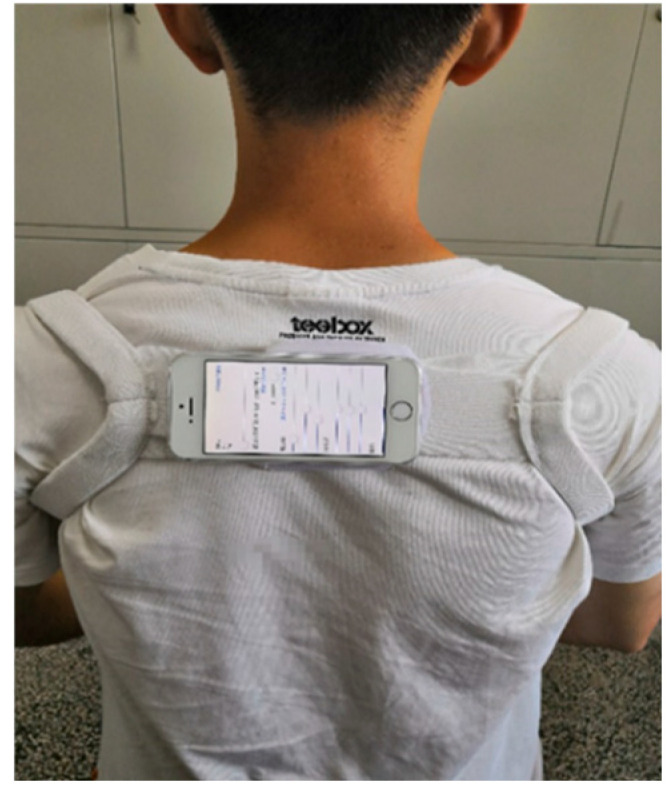
Placement of posture detection device.

The iPhone 6s Plus and iPhone SEs were used to collect the tri-axial acceleration, tri-axial angular acceleration, and three Euler angles of the vehicle and three passengers, respectively. The iPhone 6s Plus was fixed on the dashboard of the vehicle, and the iPhone SE was fixed on the upper back of the participant (Figure 2). The built-in sensors of the mobile phone were InvenSense MP67B, accelerometer full frame sensing range of ±2 g, ±4 g, ±8 g and ±16 g, gyroscope full frame sensing range of ±250, ±500, ±1000, ±2000 °/s, and the motion parameter acquisition software was Acceleration 1.1, with a sampling frequency of 100 Hz.

### 2.3. Experimental Site and Maneuvers

**Figure 3:**
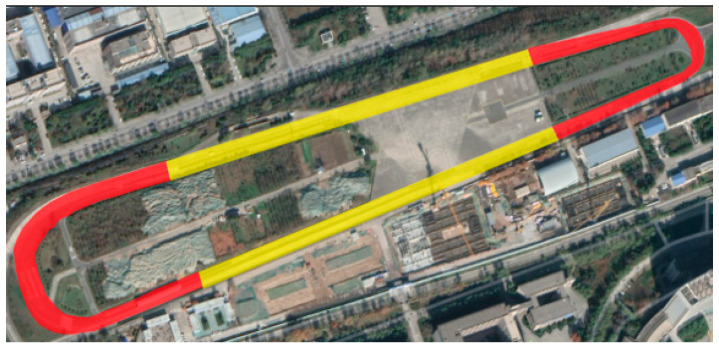
The internet of vehicles and intelligent vehicle testing grounds.

Chang’an University’s testing grounds for internet of vehicles and intelligent vehicles was selected as the test site. The track was divided into different sections (Figure 3). The driver performs turning conditions in the curve part (yellow areas) and other driving conditions in the straight part (red areas).

To simulate the daily driving conditions of an intelligent vehicle as much as possible, the trained driver performed common driving operations (the detail is showed in Table 1).

A conventional deceleration task required the driver to use the brakes to bring the car from a speed of 60 km/h to a complete stop. A conventional acceleration task required the driver to bring the car from a static state to 60 km/h. Rapid deceleration and acceleration task referred to an operation that decelerates and then accelerates, in which the vehicle’s speed was reduced from 60 km/h to 30 km/h and then immediately accelerated back to 60 km/h. A lane-change task consisted of accelerating to 60 km/h, changing the lane while holding the speed stable, and continuing to drive at the same stable speed in the target lane. In the turning task, the turning speeds were 20 km/h, 30 km/h, and 40 km/h, respectively.

### 2.4. Procedure

The purpose of the experiment was explained to the subjects, and they were asked to fill out the informed consent form. Subjects were informed that they could maintain their daily sitting posture throughout the experiment. Then the posture detection device was placed on the subjects, and a handheld comfort evaluation device was handed to them. The test was initiated after ensuring that the subjects could use the comfort evaluation device as required. During the test, the driver was required to operate the vehicle according to his own driving habits and the maneuver commands from the experiment staff. After each operation, the driver reminded the subjects to evaluate their comfort based on their feelings. There was a smooth ride of the vehicle between each maneuver. Each test lasted approximately 30 min and included 9 lane-changing maneuvers, 18 turning maneuvers, 9 conventional acceleration maneuvers, 9 conventional deceleration maneuvers, and 9 rapid deceleration and acceleration maneuvers. A single test duration of approximately 30 min was chosen to minimize fatigue while still capturing the onset and short-term progression of comfort/discomfort during repetitive manipulations, supported by preliminary testing and relevant research [26,27,28]. At the same time, the order of driving operations was completely randomized for the participants.

### 2.5. Data Preprocessing Techniques

The time error between the different mobile phones was calculated based on the vehicle motion data collected during the test by the iPhone 6s Plus and the time corresponding to the acceleration peak values collected by the phone before the test. Thus, the time of the two iPhones SE collecting the posture data of the subjects was calibrated. Prior to formal testing, we extended this procedure into a full clock synchronization and sampling verification protocol: all three phones simultaneously captured a rapid acceleration–deceleration maneuver, and we extracted each device’s peak-event timestamps to compute and correct initial clock offsets before every run. We also performed short (≈5 min) static and dynamic pre-checks, which verified that inter-sample intervals clustered tightly around 10 ms with no dropped frames or abnormal jitter, to ensure stable 100 Hz sampling.

To ensure measurement accuracy and comparability across devices and subjects, the following preprocessing and calibration steps were applied to all posture and vehicle signals. First, a brief stationary period was recorded immediately before each run and used to estimate and remove static sensor bias (zero-offset) on each axis. Second, gravity-based orientation alignment was performed during the stationary period to establish each device’s local vertical (z) axis as an initial orientation reference. Third, subject-frame measurements were rotated into the vehicle coordinate frame via a direction-cosine-matrix (DCM) transform [29], eliminating errors due to differences in how devices were mounted on individual subjects. Finally, the “coif4” wavelet function in MATLAB R2018b was used to denoise both vehicle-operation signals and passenger posture swings, a common processing step for driving data [30]. These synchronization, calibration, coordinate-transformation and denoising steps together ensure that the derived posture features are temporally aligned with vehicle kinematics and robust to device offsets and mounting variability.

### 2.6. Passenger Comfort Prediction Model

In this study, passenger comfort prediction models were established based on traditional machine learning and deep learning algorithms.

#### 2.6.1. Passenger Comfort Prediction Model Based on Dynamic Time Warping (DTW) and K Nearest Neighbor (KNN)

The distance measurement of the KNN algorithm is generally the Euclidean distance, but the standard Euclidean distance will become inaccurate in the face of multi-attribute feature space division. Therefore, this paper integrates the DTW method as the distance measurement of the KNN algorithm to improve the accuracy of the classification results [31]. Given two sequences ${\left\{x_{i}(t)\right\}}_{t=1}^{T}$ and ${\left\{x_{j}(u)\right\}}_{u=1}^{U}$, the DTW distance is:

(1) $$D_{\mathrm{DTW}}(i,j)=\underset{w\in W}{\min}\sum_{(t,u)\in w}\left\|x_{i}(t)-\right.{\left.x_{j}(u)\right\|}_{2}$$

where W denotes all valid warping paths.

Integrating the previous statistical inference and ridge regression analysis results, when establishing the deep learning model, the selected passenger posture instability data include pitch angle, roll angle, pitch angle velocity, roll angle velocity, pitch angle acceleration, roll angle acceleration, longitudinal acceleration and lateral acceleration. Since the dimensions of the instability parameters of the passenger’s posture are different, and the range of change is also different, it is necessary to standardize the characteristic parameters.

(2) $$x^{\prime }=\frac{x-\mu_{x}}{\sigma_{x}}$$

where $\mu_{x}$ and $\sigma_{x}$ are its sample mean and standard deviation.

In addition to the passenger’s posture instability, the passenger’s personal characteristics are also input into the model as feature parameters, which mainly include gender and motion sickness susceptibility. In addition, based on the above analysis results, it can be seen that in different driving conditions, the posture of passengers is not exactly the same. In order to ensure the accuracy of the model, the type of driving conditions is also input into the model as characteristic parameters. Because five driving conditions are considered in this paper, which belong to multi classification label, the One-hot encoding method is used to convert the driving operation classification labels into binary vector representations. Classification is then performed by KNN using DTW distances: for a test sequence i, compute $D_{\mathrm{DTW}}(i,j)$ to all training sequences j, select its k nearest neighbors $N_{k}(i)$ and predict the comfort class by majority vote.

#### 2.6.2. BiLSTM with the Attention Mechanism

The BiLSTM neural network model is a new model formed by combining the advantages of the bidirectional recurrent neural network (RNN) and LSTM models, and it has been successfully applied in many time-series tasks. The attention mechanism algorithm can filter the most important part from a large amount of information, thereby improving the calculation speed and accuracy of the algorithm. Therefore, the model of passenger comfort proposed in this paper incorporates the attention and BiLSTM models.

The established model includes an input layer, a BiLSTM layer, an attention layer, and an output layer. The masking layer was selected as the input layer. The main function of the masking layer is to “shield” specific input time-series data, and to locate and skip specific time steps. The BiLSTM layer is used to perform preliminary feature extraction on the input data. The role of the attention layer is to perform linear weighting on the data output by the BiLSTM layer. Passenger comfort judgment is a two classification problem; thus, sigmoid, Adam, and binary_crossentropy were, respectively, selected as the activation function, optimizer, and loss function.

For the BiLSTM layer, we use standard LSTM cell equations for each direction. For a single-direction LSTM at time t, the updates are

(3) $$i_{t}=\sigma (W_{i}x_{t}+U_{i}h_{t-1}+b_{i})$$

(4) $$f_{t}=\sigma (W_{f}x_{t}+U_{f}h_{t-1}+b_{f})$$

(5) $$o_{t}=\sigma (W_{o}x_{t}+U_{o}h_{t-1}+b_{o})$$

(6) $${\tilde{c}}_{t}=\tanh(W_{c}x_{t}+U_{c}h_{t-1}+b_{c})$$

(7) $$c_{t}=f_{t}⊙c_{t-1}+i_{t}⊙{\tilde{c}}_{t}$$

(8) $$h_{t}=o_{t}⊙\tanh(c_{t})$$

where σ(⋅) is the sigmoid function and ⊙ denotes element-wise multiplication. The forward and backward hidden states are concatenated to form the BiLSTM representation:

(9) $$h_{t}^{\mathrm{bi}}=[{\vec{h}}_{t};{\overset{\leftarrow }{h}}_{t}]\in {\mathbb{R}}^{2H}$$

where H is the hidden size of one-directional LSTM.

We compute a scalar score $e_{t}$ for each time step using a score function s(⋅), convert these scores into normalized attention weights $\alpha_{t}$ via softmax, and form a context vector c as the weighted sum of BiLSTM outputs:

(10) $$e_{t}=s(h_{t}^{\mathrm{bi}})$$

(11) αt=exp(et)∑T∑k=1Texp(ek)

(12) $$c=\sum_{t=1}^{T}\alpha_{t}h_{t}^{\mathrm{bi}}$$

Static features (such as gender, motion-sickness susceptibility, and driving-condition one-hot vectors) may be concatenated with c and passed to a final fully connected layer. For binary classification we use a sigmoid activation:

(13) $$\hat{y}=\sigma (W_{o}[c;s]+b_{o})$$

where s denotes static features and $\hat{y}$ denotes the predicted probability of discomfort.

Training minimizes binary cross-entropy with L2 regularization:

(14) $$L=-\frac{1}{N}\sum_{i=1}^{N}\left(y^{\left(i\right)}\log{\hat{y}}^{\left(i\right)}+(1-y^{\left(i\right)})\log(1-{\hat{y}}^{\left(i\right)})\right)+\lambda {\left\|\Theta \right\|}_{2}^{2}$$

## 3. Results

A total of 3240 posture samples were collected, and 5 samples had missing data, so the final data used for analysis was 3235. The longitudinal and transverse acceleration, the pitch and roll angle, as well as the pitch and roll angular velocity of passengers were selected as posture swing parameters. Specifically, the posture instability parameters were determined based on the driving condition, and all parameters were taken as the maximum absolute value of each in one driving condition.

### 3.1. Qualitative Research on Posture Instability and Subjective Comfort

In order to compare the differences in postural swing of passengers with different subjective comfort ratings, the samples that experienced the same vehicle motion but with different subjective comfort ratings and had the same subject traits (gender and susceptibility to motion sickness) were selected. Then, the paired sample t-test method, in which the test level was set to 0.05, was used for variance analysis. The relationship between posture instability and subjective comfort was studied in relation to both longitudinal and lateral vehicle control.

#### 3.1.1. Vehicle Longitudinal Control Conditions

Under the conventional acceleration condition, 88 samples were found to be inconsistent with the subjective comfort evaluation of two passengers under the same driving condition, and 46 samples conformed to the principle of homogeneity of the subjects. To study the influence of posture instability on comfort during conventional acceleration, the selected parameters were the longitudinal acceleration, pitch angular velocity, and pitch angle of the passenger.

**Figure 4:**
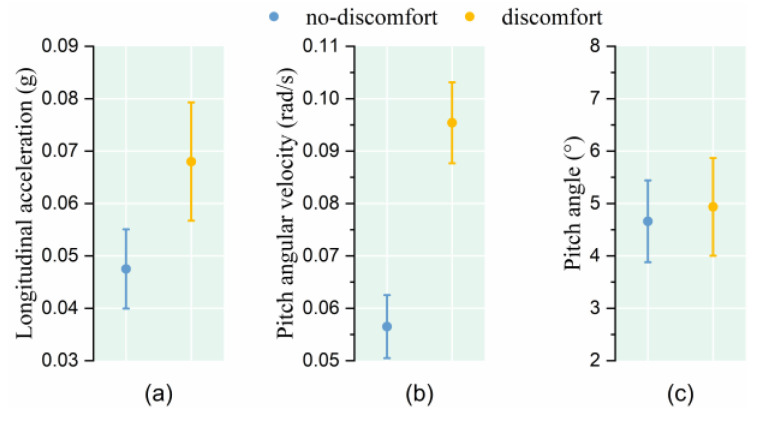
Relationship between passenger posture instability and comfort level under conventional acceleration. (**a**) Longitudinal acceleration; (**b**) Pitch angular velocity; (**c**) Pitch angle.

The results demonstrate that when facing the same vehicle acceleration, the passengers who indicated discomfort experienced a higher longitudinal acceleration and pitch angular velocity, while the pitch angle was basically the same (as shown in Figure 4 and Table 2).

Under the conventional deceleration condition, 82 samples were found to be inconsistent with the subjective comfort evaluation of two passengers under the same driving condition, and 46 samples conformed to the principle of homogeneity of the subjects. The posture parameters considered during the conventional deceleration conditions were longitudinal deceleration, pitch angular velocity, and pitch angle.

**Figure 5:**
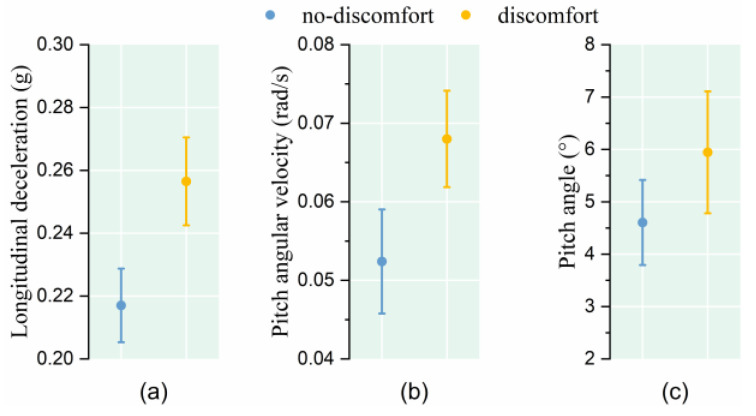
Relationship between posture instability and comfort level under conventional deceleration. (**a**) Longitudinal deceleration; (**b**) Pitch angular velocity; (**c**) Pitch angle.

The research results show that, facing the same vehicle deceleration stimulus, the longitudinal deceleration and pitch angular velocity of passengers who indicated discomfort were greater, but there was no significant difference in the pitch angle (as shown in Figure 5 and Table 2).

Under the rapid deceleration and acceleration driving condition, 147 samples were found to be inconsistent with the subjective comfort evaluation of two passengers under the same driving condition, and 92 samples conformed to the principle of homogeneity of the subjects. The passenger longitudinal acceleration, longitudinal deceleration, longitudinal acceleration and deceleration amplitude, passenger pitch angle velocity and passenger pitch angle were selected for exploring the passenger posture swing in the rapid deceleration and acceleration.

**Figure 6:**
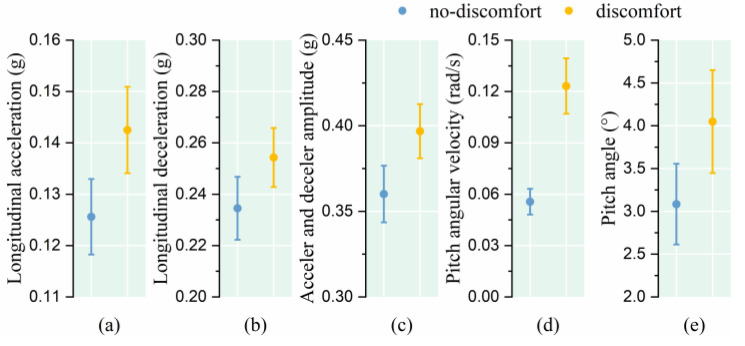
Relationship between posture instability and comfort level under rapid deceleration and acceleration. (**a**) Longitudinal acceleration; (**b**) Longitudinal deceleration; (**c**) Acceleration and deceleration amplitude; (**d**) Pitch angular velocity; (**e**) Pitch angle.

The research results show that, facing the same vehicle rapid deceleration and acceleration stimulus, the longitudinal acceleration, longitudinal deceleration, longitudinal acceleration and deceleration amplitude, pitch angular velocity, and pitch angle of passengers who indicated discomfort were greater (as shown in Figure 6 and Table 2).

#### 3.1.2. Vehicle Lateral Control Conditions

**Figure 7:**
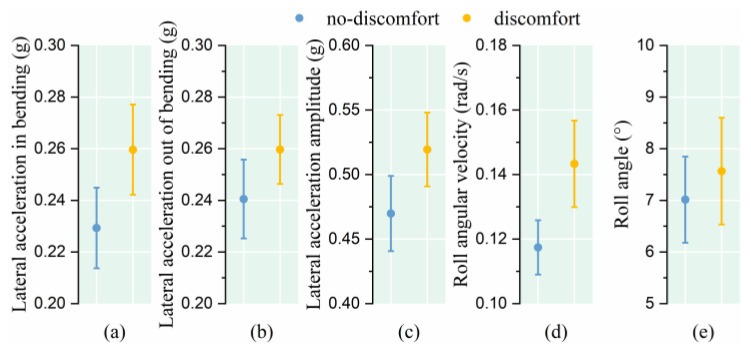
Relationship between posture instability and comfort level under turning condition. (**a**) Lateral acceleration in bending; (**b**) Lateral acceleration out of bending; (**c**) Lateral acceleration amplitude; (**d**) Roll angular velocity; (**e**) Roll angle.

Under the vehicle turning condition, 136 samples were found to be inconsistent with the subjective comfort evaluation of two passengers under the same driving condition, and 64 samples conformed to the principle of homogeneity of the subjects. In the study of passenger posture swing during turning conditions, the passenger lateral acceleration in bending, lateral acceleration out of bending, lateral acceleration amplitude, roll angle velocity and roll angle were selected. The results show that, when facing the same vehicle lane-changing stimulus, the passengers who indicated discomfort experienced higher lateral acceleration and a higher pitch angular velocity (as shown in Figure 7 and Table 2).

Under the vehicle lane-changing condition, 174 samples were found to be inconsistent with the subjective comfort evaluation of two passengers under the same driving condition, and 85 samples conformed to the principle of homogeneity of the subjects. A paired sample t-test was performed on 85 groups of data. The lane-changing condition included both lateral and longitudinal vehicle motions, so that both lateral and longitudinal attitude parameters were considered in this condition.

**Figure 8:**
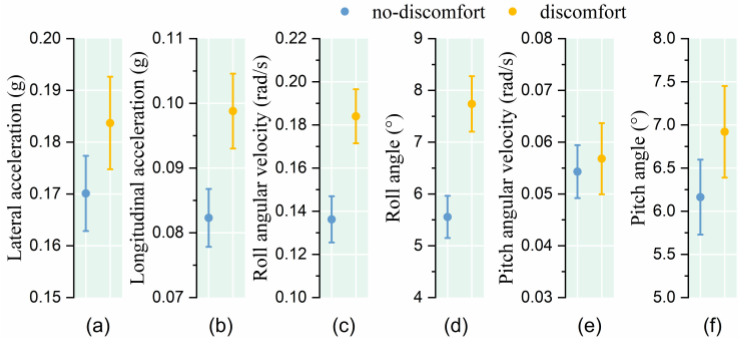
Relationship between posture instability and comfort level under lane-changing condition. (**a**) Lateral acceleration; (**b**) Longitudinal acceleration; (**c**) Roll angular velocity; (**d**) Roll angle; (**e**) Pitch angular velocity; (**f**) Pitch angle.

The results show that, in the face of the same vehicle turning stimulus, passengers who indicated discomfort experienced greater body lateral acceleration, roll angular velocity, and roll angle, but there was little difference in the pitch angular velocity and pitch angle (as shown in Figure 8 and Table 2).

For the comprehensive comparison and analysis of the difference in the degree of posture sway between passengers with different comfort ratings in different driving conditions, the results are presented in Table 2. It can be seen that during the same vehicle motions, uncomfortable passengers experienced a greater posture swing. When experiencing longitudinal driving conditions, passengers who selected “discomfort” experienced higher longitudinal acceleration and pitch angular velocity than those who selected “no-discomfort.” When experiencing rapid vehicle deceleration and acceleration, the pitch angle of the passengers who selected “discomfort” was also higher. In lateral driving conditions, the lateral acceleration and roll angular velocity of the passengers who selected “discomfort” were higher than those of the passengers who selected “no-discomfort.” During the combined lateral and longitudinal motion of the vehicle (lane-changing condition), the lateral and longitudinal accelerations of passengers who evaluated discomfort were higher than those who selected “no-discomfort.” and the roll angle and its angular velocity of the passengers who selected “discomfort” were also higher than those of the passengers who selected “no-discomfort.”

### 3.2. Quantitative Analysis of the Influences of Posture Instability Parameters on Comfort

In Section 3.1, the relationship between passenger posture instability and comfort was proven. In this section, the internal mechanism affecting passenger comfort was explored by modeling the weighting coefficients of different posture parameters on comfort. Regarding the internal mechanism of comfort, the perception ability of the human perception system must be further considered.

Research shows that the main motion-sensing system of the human body is the vestibular system, which contains two organs, namely the semicircular canal and otoliths. The semicircular canal is primarily used to measure the angular velocity changes in the human body. Therefore, in the selection of parameters, in addition to the angular velocity, the change in the angular velocity must also be considered; thus, the standard deviation of the human angular velocity is considered in this section. The otoliths can sense the current acceleration of the human body, as well as the direction of acceleration. Therefore, human acceleration should be considered when analyzing the internal mechanism of comfort.

Ridge regression is a biased estimation method, the accuracy of which is much better than that of unbiased estimation when facing the multi-collinearity problem of independent variables. Therefore, ridge regression was employed in the present study to establish the relationship model between passenger posture instability parameters and comfort.

Taking conventional acceleration as an example, ridge regression looked at the passenger pitch angular velocity, the standard deviation of pitch angular velocity, and longitudinal acceleration in analyzing the relationship between the posture instability and comfort. Passenger gender and carsickness susceptibility were also reflected in the model.

**Figure 9:**
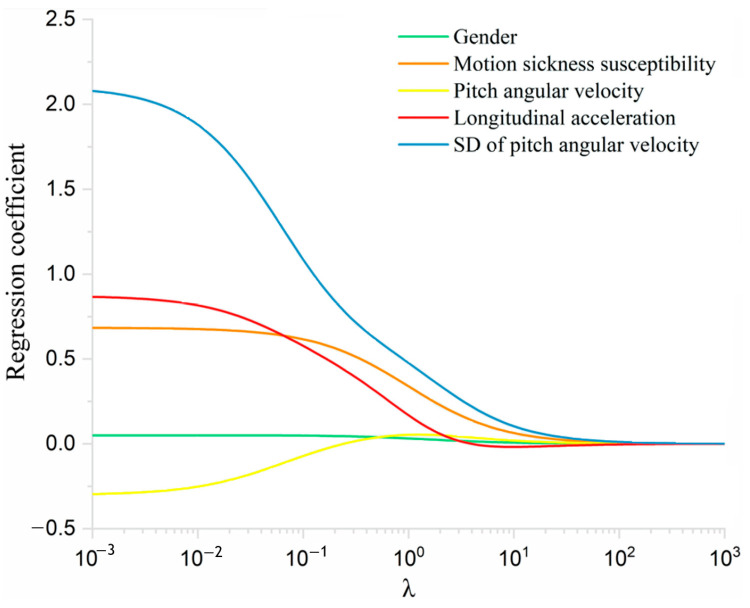
Ridge trace of postural instability parameter coefficients with ridge coefficients and optimal λ under acceleration.

**Figure 10:**
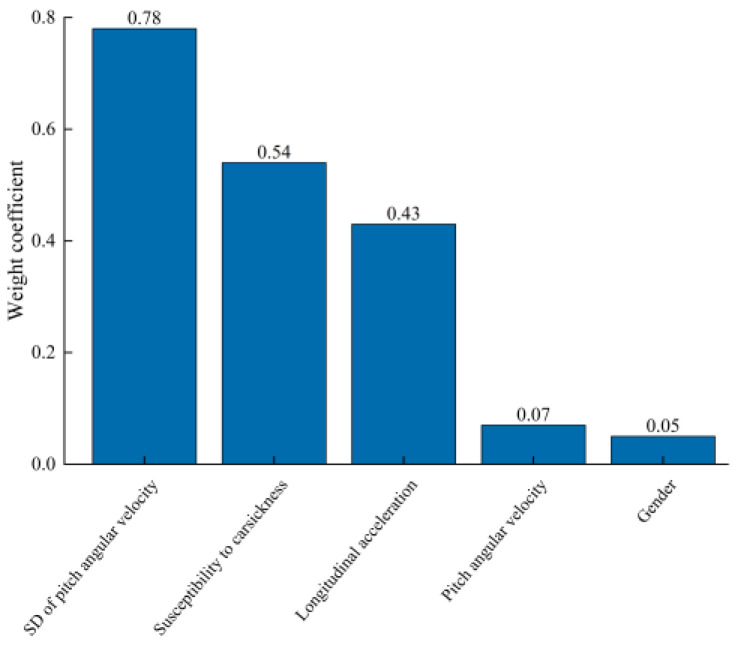
Weight coefficients of posture instability during conventional acceleration.

Figure 9 shows the relationship between the regression coefficients of the respective variables and the ridge coefficient. It can be seen that when the ridge coefficient is between 0.01 and 1, all of the coefficients begin to shrink. The optimal ridge coefficient λ = 0.25 and MSE = 0.35 were calculated through the 10-fold cross-validation. Substituting λ = 0.25 into the ridge regression (as shown in Figure 10) yields relatively large coefficients for the standard deviations of longitudinal acceleration and pitch angular velocity (0.43 and 0.78, respectively), indicating that these two measures are the primary contributors to passenger discomfort under acceleration.

**Figure 11:**
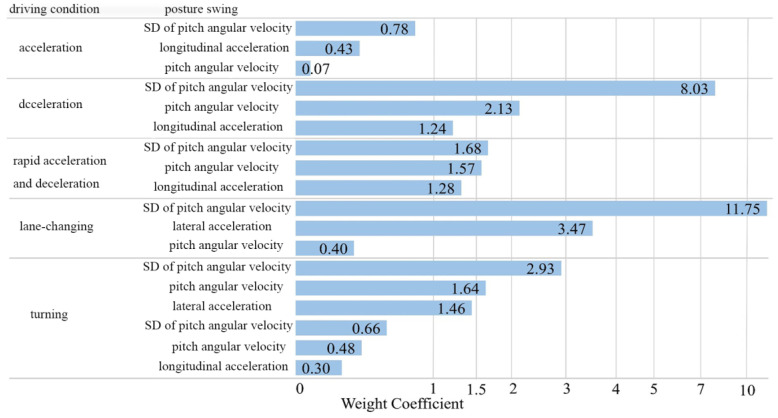
Weights coefficient of the influences of posture instability parameters.

Ridge regression was also employed for the other driving conditions, and the results are shown in Figure 11. It can be seen that the posture parameters that have a significant impact on passenger comfort vary under different driving conditions. In general, under the three longitudinal driving conditions, the weight coefficient of the standard deviation of the pitch angular velocity was found to be higher than those of the other instability parameters, indicating that excessive changes in the pitch angular velocity can reduce passenger comfort. Similarly, under the lateral driving conditions, the weight coefficient of the standard deviation of the angular velocity was found to be higher than those of the other instability parameters, indicating that passenger comfort is more affected by the change in the roll angular velocity. The results show that passenger comfort is more sensitive to changes in angular velocity than to changes in acceleration.

### 3.3. Passenger Comfort Prediction Model Results

The above sections verified that posture instability is the cause of passenger discomfort and identified the posture parameters that have an impact on passenger comfort, as well as the degree of impact of each parameter. This study evaluates the constructed passenger comfort prediction models. The specific evaluation results of each model are presented below.

#### 3.3.1. Passenger Comfort Prediction Model Based on Dynamic Time Warping (DTW) and K Nearest Neighbor (KNN) Results

The full data set was divided into a set of 2588 training samples and a set of 647 test samples. The training samples were further divided into a training set (2070 samples) and a validation set (518 samples). The input parameters of the model included a passenger rotation angle, angular velocity, angular acceleration, linear acceleration, driving condition type, and passenger personal characteristics.

**Figure 12:**
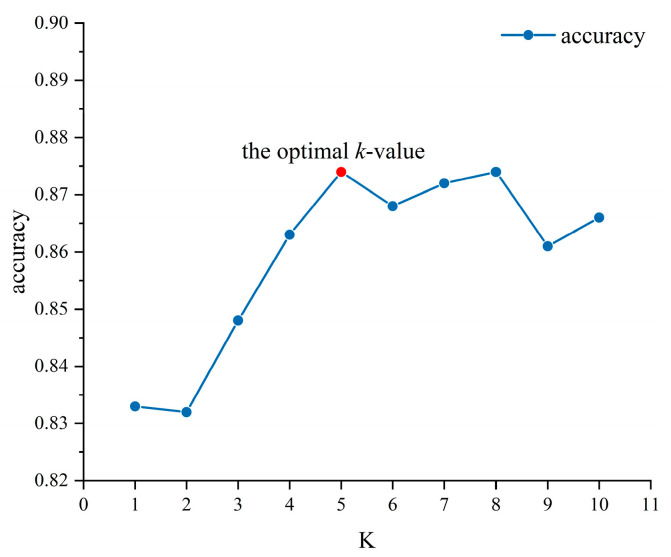
The selection of the k-value.

Model Evaluation. In the DTW + KNN classification algorithm, the optimal k-value was determined via 10-fold cross-validation (As shown in Figure 12). When evaluating the prediction effect of the model, in addition to the accuracy of the test set, the recall, precision, and F1-score were also considered.

**Figure 13:**
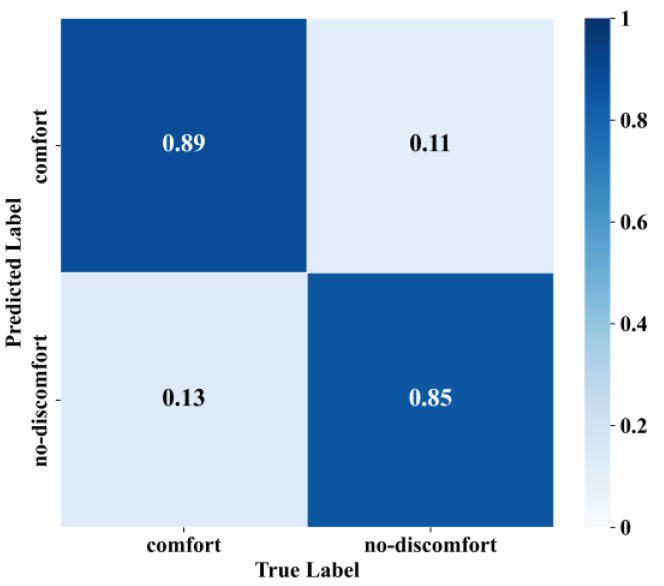
The test set confusion matrix.

The optimal k-value was introduced into the model to predict the test set data, and Figure 13 presents the confusion matrix of the DTW + KNN model for the prediction of the test set data. The overall prediction accuracy of the model was 87.1%; it could correctly judge 89.3% of passenger comfort, while the accuracy of identifying passenger discomfort was 84.8%.

#### 3.3.2. BiLSTM with the Attention Mechanism Results

**Figure 14:**
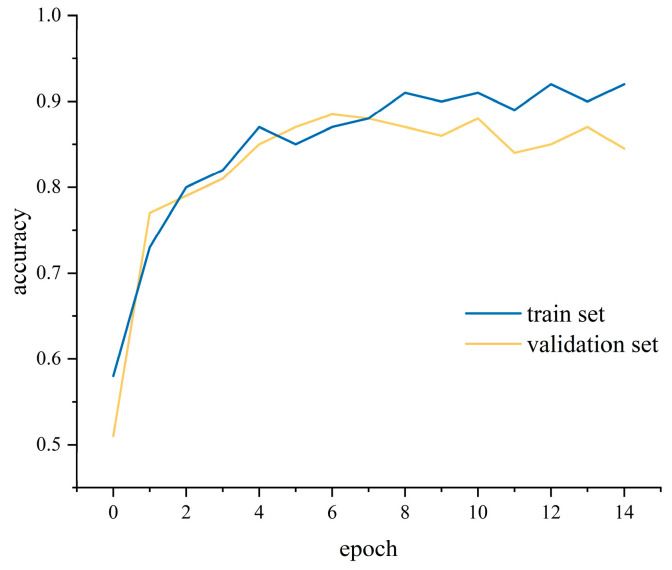
The model accuracy of the training set.

**Figure 15:**
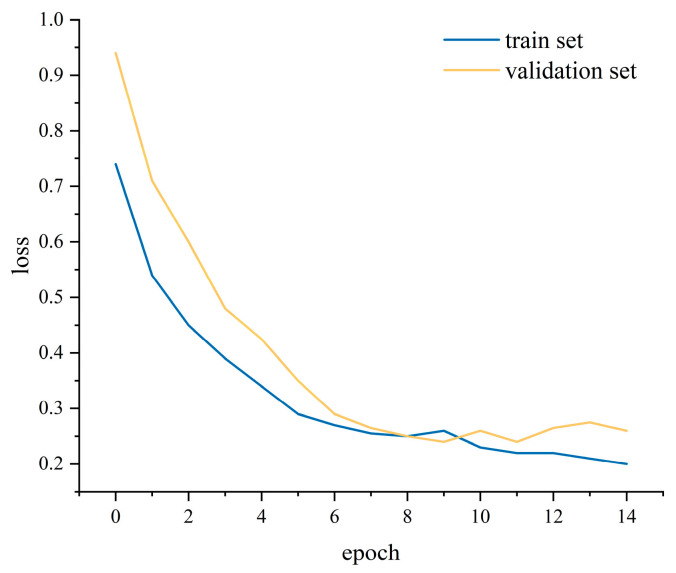
The loss value of the training set.

**Figure 16:**
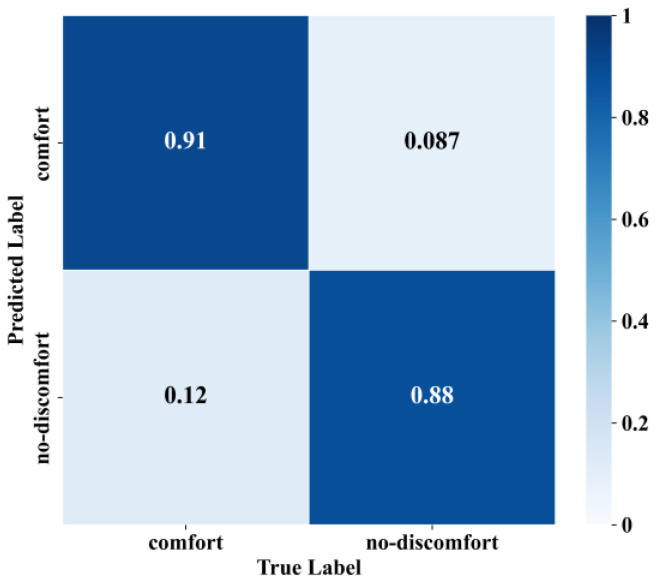
The test set confusion matrix.

During training, we monitored both loss (Figure 14) and accuracy (Figure 15) on the training and validation sets and applied early stopping when validation loss failed to improve for three consecutive epochs—this triggered at the ninth epoch, balancing rapid convergence and generalization. To mitigate overfitting, a dropout layer was inserted between the BiLSTM and attention modules, and L2 weight decay was applied in the optimizer. Key hyperparameters, including the number of hidden units, learning rate, and batch size, were selected via grid search to ensure concurrent improvements in both training and validation performance. The model was used to judge the samples of passenger posture instability in the test set, and the results are exhibited in Figure 16 and Table 3. It can be seen that the model judged the “no discomfort” state of passengers with 91% accuracy and the “discomfort” state with 88% accuracy, thereby maintaining the overall accuracy rate of the model at 89%. The model can ensure high accuracy and a high F1-score, and can effectively judge the subjective comfort of passengers based on the extent of their posture instability.

Via model comparison (Table 3), it is evident that the deep learning model based on the BiLSTM network and the attention mechanism exhibited a better predictive effect on passenger comfort; the overall accuracy of the model reached 89%, which can be attributed to the improvement of the intelligent driving vehicle control algorithm, thereby improving the vehicle ride comfort.

## 4. Discussion

The popularization of self-driving vehicles can improve road safety, reduce environmental pollution, alleviate traffic congestion, and even free passengers from restrictions (self-driving cars alleviate concerns about needing a driver’s license, being too old, driving intoxicated, or becoming distracted). It is expected that when self-driving vehicles replace traditional vehicles, the traffic accident rates will be greatly reduced, and public resources will be conserved. However, public acceptance is an important challenge to the popularization of autonomous vehicles. It is well known that passenger comfort is strongly related to acceptance [2,5,25], so improving the comfort of autonomous vehicles can increase their popularity.

At present, the technology of automatic driving and vehicle road coordination has become the focus of competition in the world. However, whether it is the ITS strategic plan of the United States, the INFRAMIX_2_ project of Europe or the HORIZON 2020 Project of China, more attention is paid to the safety problem, and less attention is paid to the comfort of intelligent vehicles. In this research, an experiment was carried out based on a trained driver driving a traditional vehicle and 60 participants took part in. During the test, the driver simulated the working conditions that an intelligent vehicle may perform in daily driving. In addition to conventional driving conditions, such as conventional acceleration and deceleration, turning, and lane-changing, rapid deceleration and acceleration were included in this study. In the daily traffic in China, vehicles often decelerate when approaching a crosswalk or intersection, and accelerate immediately after passing. The rapid deceleration and acceleration conditions were set in this study to simulate this situation. Moreover, under these conditions, the sign of the acceleration of the vehicle under the rapid deceleration condition will change. Research by Hu et al. [32] revealed that passenger discomfort is more likely to be caused during vehicle movement with changes in acceleration signs. To increase the experimental reliability, during the test, the driver informed the subjects of the upcoming operations in advance, thereby preventing passengers from experiencing perception conflict. In the experiment, the subjects’ posture data were collected via smartphones, and the subjects’ subjective comfort data were collected via real-time acquisition equipment, thereby achieving the real-time collection of the passengers’ subjective comfort data and objective posture instability data.

Many studies have demonstrated the correlation between passenger posture instability and carsickness, i.e., people prone to carsickness exhibit more posture instability [8,33,34]. In this study, the relationship between posture instability and passenger subjective comfort was explored. Based on the results of the paired sample t-test, it was found that under the same driving conditions, passengers with different experiences of comfort reported significantly different posture instability. Also, the speed of posture instability (including the lateral and longitudinal acceleration, roll, and pitch angular velocity) was significantly higher for passengers who experienced discomfort, which verifies that the cause of discomfort is posture instability.

Studies have demonstrated the influences of gender [22,35,36] and motion sickness susceptibility [22] on passenger motion sickness, which is a type of serious discomfort. Therefore, the ridge regression model also analyzed the impacts of passenger personal characteristics (gender and carsickness susceptibility) on passenger comfort. Regarding the impacts of personal characteristics on passenger comfort, the results of this study are consistent with previous studies in that gender and susceptibility to carsickness were found to have a certain impact on passenger comfort. The current study revealed that under different driving conditions, the change in angular velocity is the main cause of passenger discomfort, indicating that discomfort is caused primarily by the signal of the semicircular canal, which further reveals the mechanism of passenger discomfort. As such, when designing intelligent driving vehicles, attention should be paid to ensure that the change in the angular velocity of the passenger’s posture remains at a relatively stable value.

## 5. Conclusions

To address the challenges of passenger comfort in autonomous vehicles and enhance public acceptance, this study focused on developing effective comfort assessment models. By integrating vehicle driving conditions, passenger posture parameters, and personal characteristics, two prediction models were developed, based on the DTW + KNN algorithm and the Attention + BiLSTM algorithm. Results showed that the DTW + KNN classifier achieved an overall accuracy of 87.1%, while the Attention + BiLSTM model achieved 89.4% on the same test set. These results demonstrate that objective postural instability features can reliably predict passengers’ subjective comfort, and that deep learning methods offer a significant accuracy advantage. The proposed model provides objective metrics and practical tools for comfort-aware motion planning in intelligent vehicles.

While the present results are promising, several limitations should be acknowledged. First, participants were aged 21–28 years, which may constrain the generalizability of our findings to drivers younger than 21 or older than 28. Second, we used a trained human driver following a scripted protocol to execute maneuvers; although this approach enabled safe, repeatable on-track testing, it does not fully replicate the lower-level control dynamics of algorithmic controllers. Third, subjective comfort was recorded with a binary device (comfortable/uncomfortable) to ensure low in-vehicle cognitive load and good temporal alignment, but this choice reduces label granularity and limits richer ordinal or continuous analyses.

To address these limitations, future work will recruit a broader age range to test robustness across life stages and validate the models under algorithmic control. This validation will involve either replaying recorded trajectories with model-based controllers or conducting comparable trials in a high-fidelity simulator to quantify any residual differences between human-executed and algorithmic maneuvers. Additionally, subjective data collection will be enriched by combining finer-grained self-report instruments (e.g., multi-level scales or continuous sliders) and physiological measures (e.g., HRV, skin temperature, head/neck kinematics) to improve label resolution and model performance. Implementing these extensions will strengthen generalizability and help translate the present predictive models into practical, comfort-aware motion planning for fully automated vehicles.

## Figures and Tables

**Table 1:** Test driving conditions.

Driving Conditions		Operation
Longitudinal Motion	Conventional acceleration	0 km/h–60 km/h
	Conventional deceleration	60 km/h–0 km/h
	Rapid deceleration and acceleration	60 km/h–30 km/h–60 km/h
Lateral Motion	Lane changing	60 km/h
	Turning	20 km/h, 30 km/h and 40 km/h

**Table 2:** The comparison of passenger posture instability parameters.

Driving Conditions	Posture Swing Index	Sig.	Estimated Marginal Mean (95% CI)		P.C.
			No-Discomfort	Discomfort	
Acceleration	Longitudinal acceleration	**	0.048 (0.032, 0.063)	0.068 (0.045, 0.092)	41.6%
	Pitch angular velocity	***	0.057 (0.044, 0.069)	0.095 (0.079, 0.111)	66.7%
	Pitch angle	NS	4.659 (3.039, 6.280)	4.935 (3.003, 6.868)	5.9%
Deceleration	Longitudinal acceleration	***	0.217 (0. 193, 0.241)	0.257 (0.228, 0.285)	18.4%
	Pitch angular velocity	*	0.052 (0.039, 0.066)	0.068 (0.056, 0.080)	30.8%
	Pitch angle	NS	4.603 (2.963, 6.243)	5.945 (3.599, 8.291)	29.2%
Rapid deceleration and acceleration	Longitudinal acceleration	***	0.126 (0.111, 0.140)	0.143 (0.126, 0.160)	13.5%
	Longitudinal deceleration	**	0.235 (0.210, 0.259)	0.254 (0.231, 0.277)	8.1%
	Acceleration amplitude	***	0.360 (0.327, 0.393)	0.397 (0.365, 0.429)	10.3%
	Pitch angular velocity	***	0.056 (0.041, 0.071)	0.123 (0.091, 0.156)	119.6%
	Pitch angle	*	3.084 (2.134, 4.034)	4.048 (2.838, 5.259)	31.3%
Turning	Lateral acceleration in bending	***	0.229 (0.197, 0.261)	0.260 (0.224, 0.295)	13.5%
	Lateral acceleration out of bending	**	0.241 (0.209, 0.272)	0.260 (0.233, 0.287)	7.9%
	Lateral acceleration amplitude	***	0.470 (0.410, 0.529)	0.519 (0.461, 0.578)	10.4%
	Roll angular velocity	*	0.117 (0.100, 0.135)	0.143 (0.116, 0.171)	22.2%
	Roll angle	NS	7.014 (5.412, 8.715)	7.566 (5.459, 6.673)	7.9%
Lane- changing	Longitudinal acceleration	***	0.082 (0.073, 0.091)	0.099 (0.087, 0.110)	23.7%
	Lateral acceleration	*	0.170 (0.156, 0.185)	0.184 (0.166, 0.202)	8.2%
	Roll angular velocity	**	0.136 (0.115, 0.158)	0.184 (0.159, 0.209)	35.3%
	Roll angle	***	5.556 (4.741, 6.371)	7.738 (6.666, 8.810)	39.3%
	Pitch angular velocity	NS	0.054 (0.044, 0.065)	0.059 (0.043, 0.071)	9.3%
	Pitch angle	NS	6.163 (5.293, 7.034)	6.920 (5.856, 7.985)	12.3%

Note: NS = no significance, * *p* < 0.05, ** *p* < 0.01, *** *p* < 0.001, P.C. = Percentage Change; Sig. = Significance.

**Table 3:** Model comparison.

Model	Accuracy	Recall	Precision	F1-Score
Attention + BiLSTM	89.4%	90.9%	88.3%	0.896
DTW + KNN	87.1%	89.3%	87.2%	0.882

## Data Availability

The data are available upon reasonable request to the corresponding author.
